# Combined Treatment with Polynucleotides and Hyaluronic Acid Improves Tissue Repair in Experimental Colitis

**DOI:** 10.3390/biomedicines8100438

**Published:** 2020-10-20

**Authors:** Giovanni Pallio, Alessandra Bitto, Antonio Ieni, Natasha Irrera, Federica Mannino, Socrate Pallio, Domenica Altavilla, Francesco Squadrito, Carmelo Scarpignato, Letteria Minutoli

**Affiliations:** 1 Department of Clinical and Experimental Medicine, University of Messina, Via C. Valeria, 98125 Messina, Italy; abitto@unime.it (A.B.); nirrera@unime.it (N.I.); fmannino@unime.it (F.M.); spallio@unime.it (S.P.); fsquadrito@unime.it (F.S.); lminutoli@unime.it (L.M.); 2 Department of Human Pathology and Evolutive Age “Gaetano Barresi”, University of Messina, Via C. Valeria, 98125 Messina, Italy; aieni@unime.it; 3 Department of Biomedical, Dental, Morphological and Functional Imaging Sciences, University of Messina, Via C. Valeria, 98125 Messina, Italy; daltavilla@unime.it; 4 Department of Health Sciences, United Campus of Malta, Msida MSD 2080, Malta; carmelo.scarpignato@gmail.com; 5 Faculty of Medicine, Chinese University of Hong Kong, ShaTin, Hong Kong

**Keywords:** IBD, colitis, polynucleotides, hyaluronic acid, CD3, CD20, CD44

## Abstract

Inflammatory bowel diseases (IBDs) are chronic conditions that can benefit from the combined treatment of adenosine receptor agonists and hyaluronic acid (HA), which, binding the CD44, has pro-survival effects. Therefore, this study investigated the effects of a mixture of polynucleotides and HA in an experimental model of dinitrobenzenesulfonic acid (DNBS)-induced colitis. A group of 40 rats received a single intra-colonic instillation of DNBS, and after 6 h, animals were randomized to receive daily: (i) saline solution; (ii) polynucleotides (Poly; 8 mg/kg); (iii) polynucleotides (8 mg/kg) plus hyaluronic acid (HA; 15 mg/kg); and (iv) hyaluronic acid (HA; 15 mg/kg). Rats in the control group (*n* = 10) received saline solution only. Seven days after induction, animals receiving Poly plus HA showed reduced clinical signs, weight loss and colon shortening, ameliorated macroscopic and histological damage, and apoptosis. Moreover, the combined treatment reduced the positivity in the colonic infiltrate of CD3 positive T cells, CD20 positive B cells and CD44. Furthermore, Poly plus HA reduced colonic myeloperoxidase activity and malondialdehyde, indicating a dampening of the inflammatory infiltrate and oxidation products. Our research demonstrated that a combined treatment of polynucleotides with hyaluronic acid had a protective effect in a model of ulcerative colitis, suggesting that this association deserves further attention for the treatment of IBDs.

## 1. Introduction

Inflammatory bowel diseases (IBDs) are inflammatory conditions of the gastrointestinal tract that mainly include ulcerative colitis (UC) and Crohn’s disease (CD). The aetiology of these diseases remains unknown, although increasing evidence suggests that IBDs could arise from altered immunological, environmental, psychological and genetic factors [1].

Ulcerative colitis is a relapsing non-transmural inflammatory disease that is restricted to the colon. Characteristic symptoms of UC are bloody diarrhoea and abdominal cramping, mainly during bowel movements. On the other hand, Crohn’s disease is a relapsing, transmural inflammatory disease of the gastrointestinal mucosa, with inflammation affecting the entire gastrointestinal tract and propagating segmentally from the mouth to the anus. The most representative clinical symptoms and signs of CD are diarrhoea, abdominal pain and fever [2].

IBDs are associated with a disequilibrium between reactive oxygen species (ROS) and the antioxidant response, giving rise to oxidative stress which is considered a key factor in the pathogenesis, progression, and severity of IBDs [3,4]. The increase in ROS stimulates the activation of nuclear factor-κB (NF-κB) with a consequent overproduction of IL-1β and TNF-α, but also the release of arachidonic acid from the oxidized membranes that is transformed into eicosanoids that further emphasize the damage and lead to an increase in adhesion molecule expression such as Intercellular Adhesion Molecule 1 (ICAM-1) and cell infiltrate, ultimately leading to epithelial cell apoptosis and mucosal damage [5]. Conventional therapy of IBDs relies on anti-inflammatory drugs, corticosteroids, immunosuppressants (e.g., methotrexate), as well as biological drugs [6]. However, the low remission rate and the severe adverse effects related to the long-term use of these therapies are not satisfactory [2]. For this reason, there is great interest in finding new therapeutic strategies with a better safety profile for the treatment of IBDs; previous experimental studies have indicated that adenosine receptor stimulation may down-regulate colonic inflammation [7].

The adenosine receptors (A1, A_2A_, A_2B_, and A_3_) are expressed on immune/inflammatory cells, such as lymphocytes, neutrophils, monocytes and macrophages [8,9], and it has been shown that stimulation of A_2A_ reduces pro-inflammatory cytokines in different disease models, including asthma, arthritis, and sepsis [10].

Besides inflammation, UC is characterized by tissue destruction and reduced repair rate, thus, compounds with healing properties could be also useful in this condition. Among the compounds that have a high safety profile and regenerative properties, hyaluronic acid (HA) is one of the most used in various pharmaceutical preparations. HA is a non-sulphated glycosaminoglycan composed of repeating disaccharide units of β-1,4-D-glucuronic acid and β-1,3-*N*-acetyl-D-glucosamine. HA has numerous wound-healing properties; in particular, it stimulates cell motility through two cell surface receptors: cluster of differentiation 44 (CD44) and hyaluronan-mediated motility receptors (RHAMM). Currently, the binding to CD44 appears to be the one that most likely directly activates intracellular signals required to stimulate processes relevant to angiogenesis [11]. Moreover, during IBD, endothelial cells display an activated phenotype and are known to express high levels of CD44 that appear to have a pivotal role for immune cell (such as monocytes, macrophages, neutrophils and lymphocytes) recruitment into inflamed tissues [12]. Since CD44 plays a role in cell migration and angiogenesis, modulation of CD44-HA binding may have profound effects on immune cell recruitment and in wound repair during IBD [13]. Therefore, it has been demonstrated that the interaction between HA and the cell surface of CD44 leads to DNA repair and survival function [14].

Previous works from our laboratories [15,16,17] have demonstrated the anti-inflammatory and tissue repair activity of a A_2A_ receptor agonist, polydeoxyribonucleotide (PDRN), that contains a mixture of polynucleotides. To investigate the effects of a combined treatment with polynucleotides and hyaluronic acid in an experimental colitis setting, this mixture was employed to improve tissue remodelling and reduce inflammation.

## 2. Material and Methods

### 2.1. Animals and Drugs

All animal procedures were in accordance with the Principles of Laboratory Animal Care (NIH publication no.85-23, revised 1985), authorised by the Ministry of Health Review Board for the care of laboratory animals (approval number 64/2017-PR 20/01/2017) and were in accordance with the ARRIVE Guidelines [18]. A total of 40 male Sprague–Dawley rats (250–300 g) were purchased from Charles River Laboratories (Calco, Milan, Italy). Animals were maintained in plastic cages under standard environmental conditions with water and food ad libitum in the Animal Facility of the Department of Clinical and Experimental Medicine of the University of Messina. After 1 week of acclimation to the facility environment, animals were randomly assigned to 4 groups of 10 animals each. Due to the high reproducibility of the chemical model that was employed and to the peculiar histological features that resembles human IBDs, it was possible to keep low the number of animals in each group [19,20].

DNBS (2,4-dinitrobenzenesulfonic acid) was purchased from Sigma-Aldrich (Milan, Italy) and dissolved in 50% ethanol. Polynucleotides and hyaluronic acid were a kind gift of Mastelli S.R.L. (Sanremo, Italy). The doses and route of administration were chosen according to previously published papers [21,22,23,24].

### 2.2. DNBS Model

Colitis was induced by DNBS administration, one of the most used experimental models to reproduce human UC [15]. In particular, 40 fasted rats were administered with a single intra-colonic instillation of DNBS (25 mg in 0.8 mL 50% ethanol) through a catheter that was inserted into the colon (for 8 cm) via the anus up to the splenic flexure. Animals were then kept for 15 min in Trendelenburg position to avoid reflux and were randomized to receive: vehicle (DNBS + Drug vehicle; 1 mL/kg; *n* = 10), polynucleotides (DNBS + Poly; 8 mg/kg, *n* = 10), polynucleotides + hyaluronic acid (DNBS + Poly + HA; 8 mg/kg; 15 mg/kg *n* = 10). As a positive control group, to demonstrate the superior effect of the combination of Poly + HA, 10 animals (DNBS + HA), received HA alone at 15 mg/kg. Treatments were administered intra-colonic starting 6 h after DNBS administration and every 24 h until the day of sacrifice. Control animals (*n* = 10) received a single intra-colonic instillation of 0.8 mL saline solution. At day 7, rats were sacrificed; the abdomen was opened by a midline incision; and the descending colon was removed, opened along the anti-mesenteric border, rinsed and cut into two equal pieces, one stored for histological assessments and the other one for measuring biochemical markers.

### 2.3. Evaluation of Body Weight and Food Intake

Body weight, food intake, stool consistency, rectal bleeding or the presence of blood in the stool were monitored daily for the duration of the study. Body weight and food intake were recorded every day between 9:00 and 10:00 a.m. from the day of induction to the end of the experiment. Body weight results were expressed as raw data and compared with food intake by using the following formula: food intake divided by body weight in grams and multiplied by 100.

### 2.4. Macroscopic Damage Score

Colon length was measured, and damage was scored by two independent observers, as previously described [25], according to the following criteria: 0 (no damage), 1 (localized hyperaemia without ulcers), 2 (linear ulcers with no significant inflammation), 3 (linear ulcers with inflammation at one site), 4 (two or more major sites of inflammation and ulceration extending 1 cm along the length of the colon), and 5–8 (one point is added for each centimetre of ulceration beyond an initial 2 cm).

### 2.5. Microscopic Damage Score

For light microscopy, colon tissues were fixed in 10% buffered formalin for 24 h. Subsequently, specimens were embedded in paraffin, sectioned at 5 μm thickness, stained with haematoxylin and eosin (H&E), and observed with a Leica microscope (Leica Microsystems, Milan, Italy) [26,27]. Assessment of tissue changes was carried out by two observers blinded to the experimental protocol. The following morphological criteria were considered: score 0 (no damage), score 1 (mild: focal epithelial oedema and necrosis), score 2 (moderate: diffuse swelling and necrosis of the goblet cells), score 3 (severe: necrosis and neutrophil infiltrate in the submucosa), and score 4 (very severe: widespread necrosis with massive neutrophil infiltrate and haemorrhage) as previously reported [15].

### 2.6. Immunohistochemical Evaluation of CD3, CD20, CD44 and Bcl-2

Paraffin-embedded tissues were sectioned (5 μm) and rehydrated, and antigen retrieval was performed by using 0.05 M sodium citrate buffer (pH 6.0) in a microwave for 5 min. Tissues were treated with 1% hydrogen peroxide to block endogenous peroxidase activity, and with normal horse serum (Vector Laboratories, Burlingame, CA, USA) to prevent nonspecific staining. Primary antibodies against either CD3, CD20, CD44 and Bcl-2 (Abcam, Cambridge, UK) were used, and the slides were kept overnight at 4 °C in a humid box. The slides were then washed in PBS, the appropriate secondary antibody was added, and the ABC system (Vectastain Elite ABC kit, Vector Laboratories, Burlingame, CA, USA) was used to detect antibody localization. The location of the reaction was visualized with diaminobenzidine tetra-hydrochloride (DAB; Sigma-Aldrich, Milan, Italy). Slides were counterstained with Mayer’s haemalum, dehydrated, and mounted with coverslips. As a part of the histologic evaluation, all slides were de-identified in regard to the treatment group and evaluated by a pathologist at 1× to 40× magnification with a Leica microscope (Leica Microsystems, Milan, Italy).

### 2.7. Measurement of Myeloperoxidase Activity

Myeloperoxidase (MPO), a marker of polymorphonuclear leukocyte accumulation, was determined as previously described [28,29,30,31]. Equal amounts of colon tissue were homogenized mechanically with the MICCRA D-1 homogenizer (Miccra Gmbh, Müllheim, Germany), in a solution containing 0.5% hexa-decyl-trimethylammonium bromide dissolved in 10 mM potassium phosphate buffer (pH 7.0). Lysates were then centrifuged for 30 min at 15,000 rpm at 4 °C. An aliquot of the supernatant was allowed to react with a solution of 1.6 mM tetra-methyl-benzidine and 0.1 mM H_2_O_2_. The absorbance was measured with a spectrophotometer at 650 nm. MPO activity was defined as the quantity of enzyme degrading 1μmol hydrogen peroxide/min at 37 °C and was expressed in units per g of tissue.

### 2.8. Malondialdehyde Measurement

The levels of malondialdehyde (MDA) in the colon were determined as an indicator of lipid peroxidation [32,33,34,35]. Equal amounts of colon tissue were homogenized mechanically with the MICCRA D-1 homogenizer; in 1.15% KCl solution, a 0.1 mL aliquot of the homogenate was added to a reaction mixture containing 0.2 mL of 8.1% SDS, 1.5 mL of 20% acetic acid, 1.5 mL of 0.8% thiobarbituric acid and 0.7 mL distilled water. Samples were boiled for 1 h at 95 °C and centrifuged at 3000× *g* for 10 min. The absorbance of the supernatant was measured by spectrophotometer at 650 nm.

### 2.9. Statistical Analysis

All quantitative data are expressed as mean ± SD for each group and compared by one-way or two-way ANOVA for non-parametric variables with Tukey post-test for intergroup comparisons. Statistical significance was set at *p* < 0.05. Graphs were drawn using GraphPad Prism software version 5.0 for Windows (GraphPad Software Inc., La Jolla, CA, USA).

## 3. Results

### 3.1. Effects of Polynucleotides and Hyaluronic Acid on Clinical Signs

**Figure 1:**
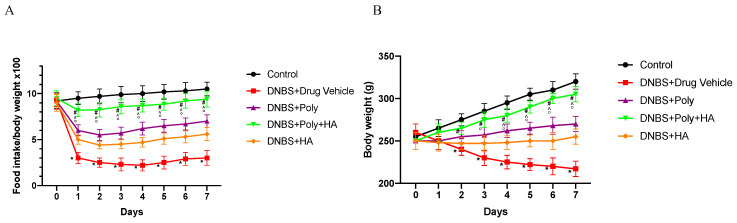
Food intake and body weight evaluated during the experimental period. Values were obtained from 10 animals per group and are expressed as means and SD. Food intake (**A**) * *p* < 0.0001 vs. control group; # *p* < 0.0001 vs. dinitrobenzenesulfonic acid (DNBS) + Drug vehicle group. Weight loss (**B**) * *p* < 0.0001 vs. control group; # *p* < 0.0001 vs. DNBS + Drug vehicle group; ° *p* < 0.001 vs. DNBS + hyaluronic acid (HA); ^ *p* < 0.001 vs. DNBS + polynucleotides (Poly).

Colitis was successfully induced in rats after DNBS administration as demonstrated by the significant loss in food intake from day 1 (*p* < 0.0001 vs. control group), followed by the appearance of diarrhoea and rectal bleeding resulting in loss in body weight from day 2 (*p* < 0.0001 vs. control group). Polynucleotides or hyaluronic acid administration slightly improved food intake and body weight. Combined treated animals demonstrated an initial reduction of weight and food intake (Figure 1A,B) followed by a complete recovery by the end of the experiment demonstrating a greater effect than polynucleotides or hyaluronic acid alone in improving the clinical features (*p* < 0.0001 vs. animals treated with DNBS + Drug vehicle; ° *p* < 0.001 vs. DNBS + HA; ^ *p* < 0.001 vs. DNBS + Poly). Reduced bleeding was observed since day 4 with the appearance of solid faeces after 2 days of treatment.

### 3.2. Effects of Polynucleotides and Hyaluronic Acid on Macroscopic Damage

**Figure 2:**
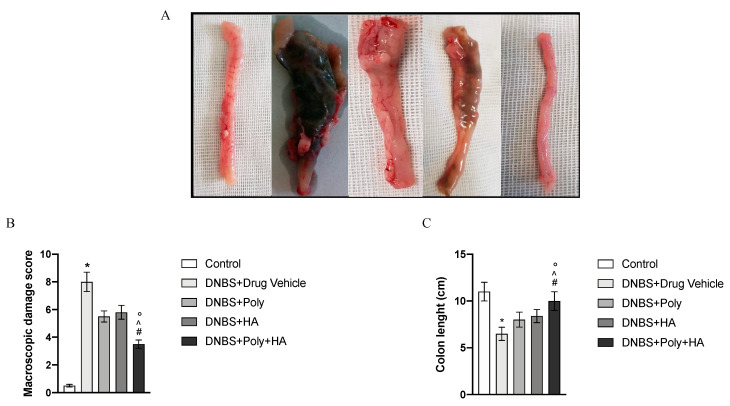
Macroscopic appearance of colon tissues (**A**) from Control (1), DNBS + Drug vehicle (2), DNBS + Poly (3), DNBS + HA (4), DNBS + Poly + HA (5). The graphs represent macroscopic damage scores (**B**) and colon length (**C**). Values were obtained from 10 animals per group and are expressed as the means and SD. * *p* < 0.0001 vs. control group; # *p* < 0.0001 vs. DNBS + Drug vehicle group; ° *p* < 0.001 vs. DNBS + HA; ^ *p* < 0.001 vs. DNBS + Poly.

At the end of the experiment, the colon of animals in the DNBS + Drug vehicle group appeared enlarged, ulcerated, oedematous, and hyperaemic compared to the control group (*p* < 0.0001; Figure 2A,B). Treatments with polynucleotides or hyaluronic acid improved this condition, and the group given the combined treatment displayed a further significant reduction in the extent and severity of colon injury, absence of ulceration, reduced size and hyperaemia (*p* < 0.0001 vs. DNBS + Drug vehicle group; Figure 2A,B). As shown in Figure 2C, DNBS also caused a significant shortening of the colon as compared to control animals (*p* < 0.0001 vs. control group) due to the tissue damage. The DNBS + Poly group and the DNBS + HA group showed an increase in colon length. Animals receiving polynucleotides plus hyaluronic acid revealed a significantly improved picture with an almost normal length of the colon, demonstrating that the effect of a combined treatment is greater than that of the two compounds alone (*p* < 0.0001 vs. DNBS + Drug vehicle group; ° *p* < 0.001 vs. DNBS + HA; ^ *p* < 0.001 vs. DNBS + Poly. Figure 2C).

### 3.3. Polynucleotides and Hyaluronic Acid Reduces Histological Damage

**Figure 3:**
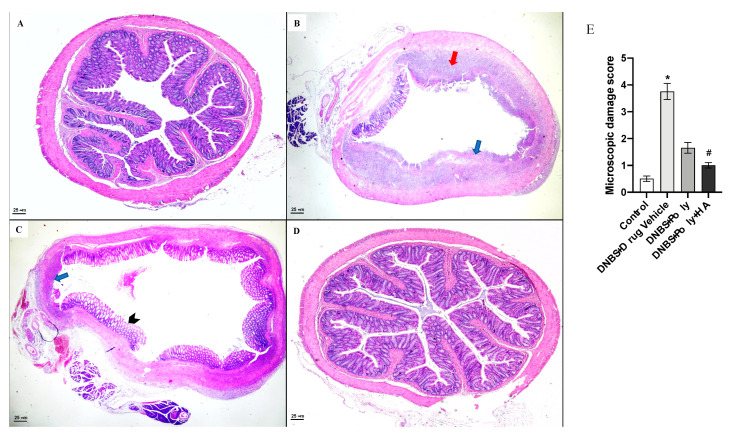
Representative haematoxylin and eosin (H&E) images (original magnification 10×) of tissues derived from control (**A**), DNBS + Drug vehicle (**B**), DNBS + Poly (**C**), DNBS + Poly + HA (**D**). The graph in (**E**) represents the microscopic damage score. Values are expressed as the means and SD of 10 animals. * *p* < 0.0001 vs. control group; # *p* < 0.0001 vs. DNBS + Drug vehicle group. The blue arrow indicates mucosal ulceration; the red arrow indicates granulation tissue; the arrow head indicates pseudopolyps.

A normal appearance of the colonic mucosa with intact epithelium was observed in the control group (Figure 3A). DNBS administration resulted in mucosal injury characterized by the following: epithelial necrosis, extensive mucosal ulceration, granulation tissue with massive infiltration of neutrophils and macrophages into the mucosal and submucosa layers, thickening of the colon wall, loss of goblet cells, transmural necrosis, oedema and crypt distortion (Figure 3B). Treatment with polynucleotides reduced the extent and severity of the histological alteration associated with DNBS administration, although residual inflamed and hyperaemic mucosa with small ulcers surrounded by pseudopolyps with mucosal bridging were observed in the DNBS + Poly treated group (Figure 3C). The combined treatment of polynucleotides plus hyaluronic acid resulted in a significant reduction in the extent and severity of epithelial and mucosal alterations associated with DNBS administration by stimulating epithelial regeneration. Colons from the DNBS + Poly + HA group were characterized by the following: absence of active inflammation in the mucosa layer, absence of erosions, intact glandular epithelial architecture, presence of oedema and fibrosis in the lamina propria with occasional foci of lymphocytes (Figure 3D).

### 3.4. Immunohistochemical Evaluation of CD3, CD20, CD44 and Bcl-2

**Figure 4:**
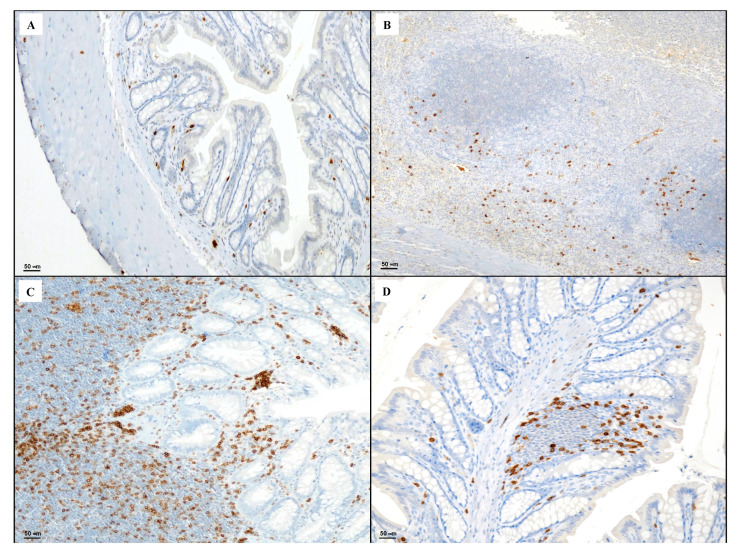
Representative CD3 immunostaining (original magnification ×20) of tissues derived from control (**A**), DNBS + Drug vehicle (**B**), DNBS + Poly (**C**), and DNBS + Poly + HA (**D**).

Tissues taken from the control group showed rare CD3 positive T lymphocytes (Figure 4A). Sections obtained from the DNBS + Drug vehicle group exhibited a strong presence of CD3 positive lymphocytes in the granulation tissue compared to the control group (Figure 4B). The DNBS + Poly group showed an increase in CD3 positive elements in the regenerative areas of the lamina propria (Figure 4C). DNBS + Poly + HA showed residual CD3 positive T lymphocytes (Figure 4D), indicating a resolution of the inflammatory process.

**Figure 5:**
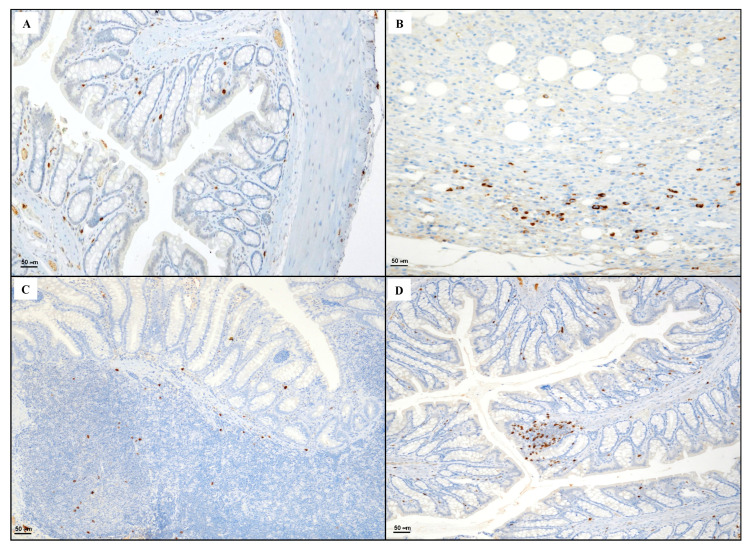
Representative CD20 immunostaining (original magnification ×20) of tissues derived from control (**A**), DNBS + Drug vehicle (**B**), DNBS + Poly (**C**), and DNBS + Poly + HA (**D**).

Section obtained from the control group showed sporadic CD20 positive B lymphocytes (Figure 5A). CD20 positive lymphocytes were detected in the granulation tissue of the DNBS + Drug vehicle group (Figure 5B). Mild immunopositivity in few lymphoid cells in the follicular and inter-follicular zone were highlighted in the DNBS + Poly group (Figure 5C), while the colon taken from the DNBS + Poly + HA group exhibited foci of CD20 positive lymphocytes in the mucosa layer (Figure 5D).

**Figure 6:**
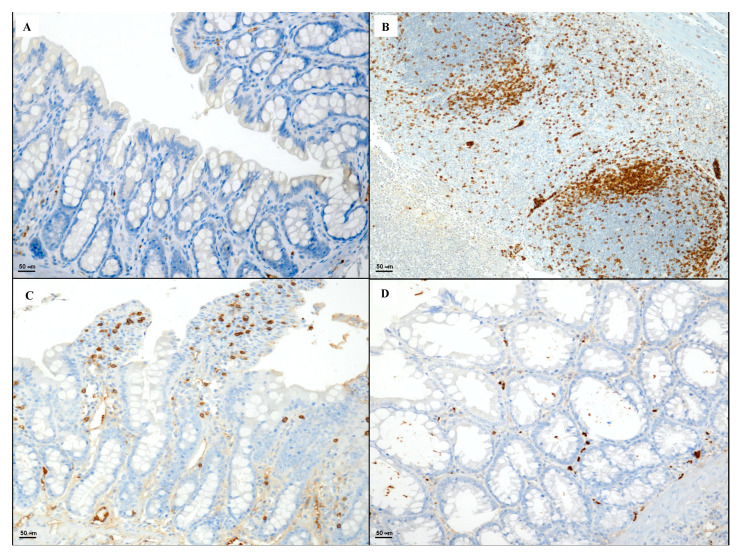
Representative CD44 immunostaining (original magnification ×20) of tissues derived from control (**A**), DNBS + Drug vehicle (**B**), DNBS + Poly (**C**), and DNBS + Poly + HA (**D**).

Colitis induced by DNBS administration caused a marked presence of CD44 positive cells when compared to the control group (Figure 6A,B). Polynucleotides administration produced a decrease in the expression of CD44 positive cells (Figure 6C). The combined treatment markedly reduced the degree of CD44 positive staining (Figure 6D).

**Figure 7:**
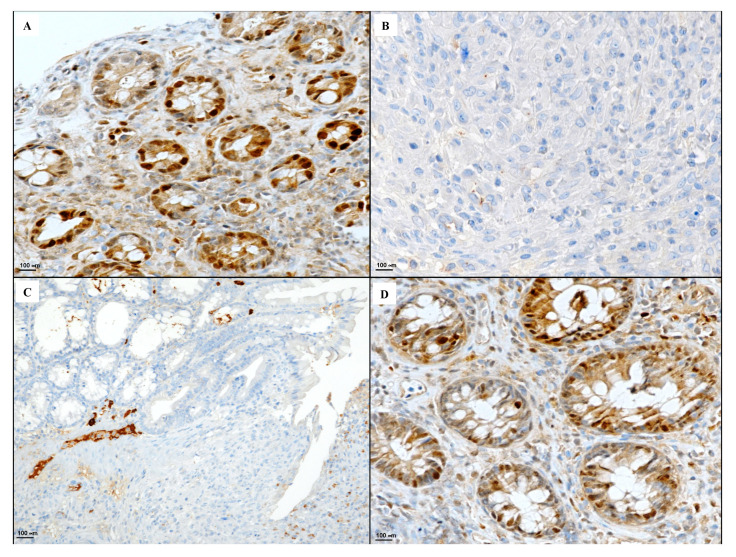
Representative Bcl-2 immunostaining (original magnification ×40) of tissues derived from control (**A**), DNBS + Drug vehicle (**B**), DNBS + Poly (**C**), and DNBS + Poly + HA (**D**).

The Bcl-2 staining in the control group showed strong cytoplasmic and nuclear immunoreactivity in epithelial cells (Figure 7A). DNBS administration abrogated the presence of Bcl-2 due to the massive necrosis of the tissue (Figure 7B). Polynucleotide treatment increased the presence of Bcl-2 in the active inflammatory area of the DNBS + Poly group (Figure 7C). Therefore, a combined treatment with polynucleotides and hyaluronic acid was able to restore Bcl-2 positivity in basal colonic-restored crypts of the DNBS + Poly + HA group (Figure 7D).

### 3.5. Effects of Polynucleotides and Hyaluronic Acid on Lipid Peroxidation and Neutrophil Infiltration

**Figure 8:**
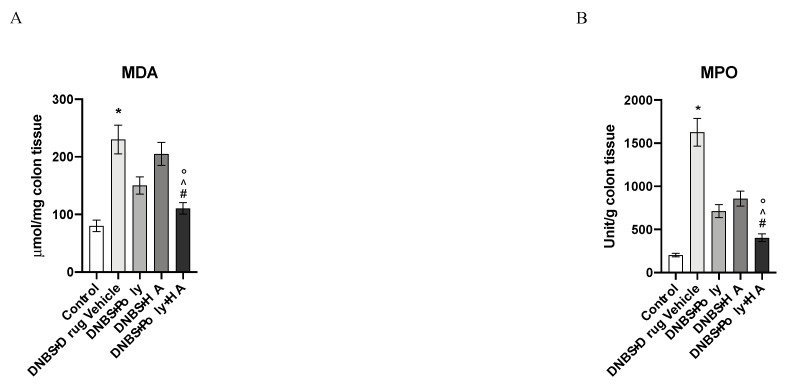
The effects of treatments on malondialdehyde levels and myeloperoxidase activity in DNBS-treated animals are shown in (**A**,**B**). All values are expressed as means and SD based on observations made on 10 animals per group. * *p* < 0.0001 vs. control group; # *p* < 0.0001 vs. DNBS + Drug vehicle group; ° *p* < 0.001 vs. DNBS + HA; ^ *p* < 0.001 vs. DNBS + Poly.

MDA levels and MPO activity were increased in the group of animals that received DNBS + drug vehicle compared to the control group (*p* < 0.0001 vs. control group; Figure 8A,B). Polynucleotides administration reduced both lipid peroxidation and the accumulation of polymorphonuclear granulocytes. In the DNBS + HA group, a reduction was observed in the myeloperoxidase activity only. The combined treatment with polynucleotides and hyaluronic acid completely blunted lipid peroxidation and MPO activity, demonstrating a greater effect compared to polynucleotides or hyaluronic acid alone (*p* < 0.0001 vs. DNBS + drug vehicle group; ° *p* < 0.001 vs. DNBS + HA; ^ *p* < 0.001 vs. DNBS + Poly. Figure 8A,B).

### 3.6. Effects of Hyaluronic Acid on Tissue Repair

**Figure 9:**
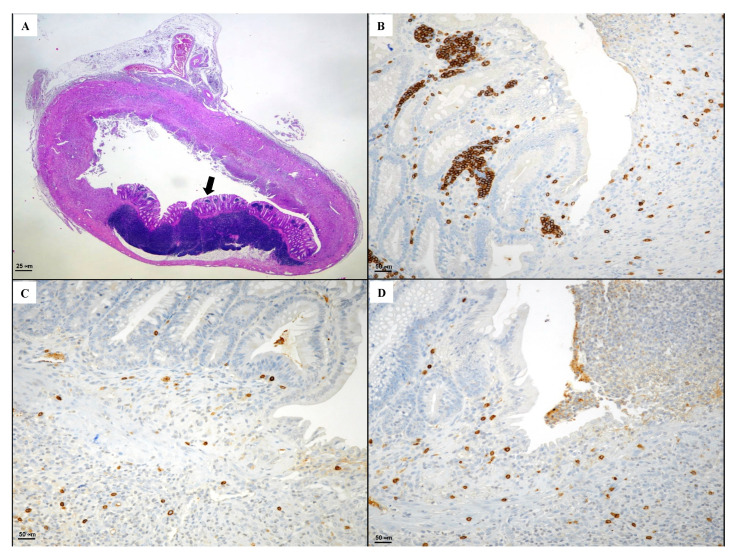
(**A**) Representative H&E images (original magnification ×10) (**B**) CD3, (**C**) CD20, (**D**) CD44 immunostaining (original magnification ×20) of tissues derived from the DNBS + HA group. The arrow indicates the regenerated area.

Considering the known beneficial effects of hyaluronic acid alone on tissue repair, a group of animals was used as positive control and following DNBS received HA. As shown in Figure 9, HA administration caused a partial mild regeneration (indicated by the black arrow). In particular, inflamed necrotic areas were evident with large mucosal ulcers, and despite the presence of healed areas, it was not possible to detect intestinal glands with mucinous phenotype. Moreover, diffuse inflammatory elements were observed in the submucosal layer, and immunostaining demonstrated a CD3 positivity in lamina propria of lymphoid cells with sporadic CD20+ and CD44+.

## 4. Discussion

In the present investigation, the beneficial effects of a combination of polynucleotides and hyaluronic acid have been tested in an experimental model of colitis and compared to the effects of HA alone. The early onset of the protective effect of the employed mixture was able to blunt the haemorrhagic diarrhoea, counterbalance weight loss and reduce the colon shortening. Moreover, it was able to restore the anatomic integrity of damaged epithelial and mucosal layers at macroscopic and histologic levels. The combined action of polynucleotides and hyaluronic acid markedly reduced the inflammatory response and granulocytic infiltration into the mucosal and submucosal layers and, as a consequence, reduced MPO activity and lipid peroxidation extent evaluated by MDA in colon samples. Therefore, combined treatment also decreased CD44 expression, usually expressed on lymphocytes and macrophages as a direct consequence of the reduction of inflammatory infiltrate. These results were confirmed by a reduced presence in the colonic infiltrate of CD3 positive T cells and CD20 positive B cells. The same results were not evident when HA or Poly alone were used; in fact, in both cases, small regenerated areas were evident with an incomplete recovery of the differentiated epithelium.

Previous papers have demonstrated that in the inflamed colonic mucosa, there is an increased apoptosis and decreased proliferation with upregulated expression of the proapoptotic Bax and downregulation of the antiapoptotic Bcl-2 proteins [15]. Treatment with the combination of polynucleotides and hyaluronic acid also affected Bcl-2 expression in experimental colitis, reducing apoptotic and necrotic cells in all tissue layers. These results confirmed that the adenosine receptor stimulation modulates the inflammatory cascade in the gastro-intestinal tract. In fact, different papers have reported the potential beneficial effects of adenosine receptor ligands, such as polynucleotides, in the management of inflammatory bowel diseases, demonstrating the efficacy of A_2A_ receptor stimulation in improving epithelial repair [7,36,37,38]. It is known that A2A is not only present on the surface of fibroblast and epithelial cells, but also on T cells and monocytes suppressing the production of specific inflammatory cytokines as IL-12, TNF-α, and IFN-γ [7]. Moreover, our results confirmed the protective effect of HA in IBD in agreement with preclinical and clinical studies previously published [11,39,40]. Additionally, the data obtained in this study support the hypothesis that this combination might be considered as a new approach since the administration of Poly + HA promoted both intestinal re-epithelization and mucosa healing during ulcerative colitis. The mechanisms by which polynucleotides and hyaluronic acid ameliorate experimental colitis are not yet fully understood, though evidence suggests that the effect may be mediated by modulation of inflammatory cell activity. In fact, macrophages are known to have an important role in the induction of tissue injury during colitis, and the activation of A_2A_ receptor has a potent macrophage-deactivating effect [41,42]. Activation of the A_2A_ receptor also modulates neutrophil function, regulates the production of reactive oxygen species by these cells [43,44], inhibits the localization of neutrophils in the endothelium by decreasing the expression of the adhesion molecules expressed on neutrophils [45], and reduces apoptosis of colonic mucosal cells [46]. Moreover, HA has an important role in reducing inflammatory responses through activation of CD44 pathways that promote the Rac-signaling cascade with improvements in DNA repair and survival function [47]. Furthermore, CD44 plays a role in cell migration and modulation of CD44–HA binding and may have profound effects on immune cell recruitment and aid in wound repair [11]. All this evidence corroborates the present findings and supports the usefulness of the Poly + HA combination. However, one of the factors that could have influenced the outcome regards HA degradation, which, in fact, could be degraded into low molecular weight fragments that could sustain inflammatory processes. In support of this theory, previous studies observed no acceleration in the recovery phase of colitis in mice receiving Low molecular weight hyaluronan hyaluronic acid (LMW-HA), thus suggesting an important role of HA size in this system [48]. How different sizes of HA mediate their differential biological effects is not well understood at a molecular level, but it is possible that LMW-HA competes for the interaction of High molecular weight hyaluronan hyaluronic acid (HMW-HA) with classical HA receptors (e.g., CD44), thus removing the anti-inflammatory protection of HMW-HA. It was also observed that a co-activator protein, TSG-6, is expressed during the healing process and cooperates with CD44 in causing the beneficial effects stimulated by HA, especially in its HMW form [40].

Some of the approved drugs used in clinical practice for IBDs, such as mesalazine, cyclosporine or biologic agents have a proven efficacy in symptom reduction, however, they are associated with the risk of developing various side effects. Therefore, there is a great need for new and cost-effective drugs with greater efficacy and tolerability, not only to ensure clinical remission but also to promote mucosal healing. In the present study, our data support the administration of a combination of Poly + HA as a new approach for promoting the intestinal re-epithelization and mucosal healing during UC. The local application of Poly + HA combination accelerated the recovery of clinical parameters and improved tissue regeneration. These results reinforced our previous findings on the effects of polynucleotide treatment in IBDs [15].

The increased visceral hypersensitivity [49] present in colitis together with the abdominal pain symptomology are particularly disabling for patients [50], thus, it is conceivable that an advantage of the polynucleotides–hyaluronic acid combination (eventually given by enema) in the therapeutic approach of UC might also reduce symptoms and not only clinical features. In a previous clinical trial, polynucleotides demonstrated a very good safety profile in patients with chronic diabetic foot ulcers [51]; thus, despite the fact that no clinical data are available regarding the efficacy of polynucleotides and hyaluronic acid in IBDs, the present data foster a potential use in UC subjects, and the topical administration amplifies the anti-inflammatory properties of both polynucleotides and HA. Since an association of polynucleotides and hyaluronic acid is currently on the market for the treatment of local dystrophies as emollient and lubricant of the anal mucosa and perianal skin, it could be readily available for clinical trials in UC patients. In this regard, preliminary pharmacokinetics data obtained in mice posit that polynucleotides have a half-life of approximately 12–17 h, whereas it has been estimated that HA’s half-life in the skin is of about 24 h [15,52], suggesting that the association of Poly + HA might be suitable for once a day dosing and easy to apply in the routine clinical practice. Nevertheless, the current study has some limitations, for instance, despite the fact that it was previously described that Poly efficacy is not related to gender [49], here, the results have been obtained in male rats only. Additionally, this experimental model well represents UC but does not provide sufficient information for treating Chron’s patients or with diffused ulcers. Moreover, we did not investigate the effects of the polynucleotides and hyaluronic acid in presence of A2_A_ and CD44 inhibitors, and we did not include a group of animals treated with infliximab or other biological agents, gold standards of IBD treatments, in order to maintain a small number of animals used in this painful procedure, according to the 3Rs principles of animal studies. Finally, we did not investigate the effects of the treatments in other colitis models such as the dextran sulphate sodium (DSS) colitis model or in other animal species, and we did not evaluate the long-term efficacy and safety of the treatment which could be an important limit considering that UC patients receive therapies spanning their life time.

In conclusion, our results revealed that the combined treatment with polynucleotides and hyaluronic acid accelerates wound healing and reduces inflammatory reaction during experimental colitis, together with a significant improvement in all pathological features.
